# Nomograms for Predicting the Incidence of Late-Onset Acute Cellular Rejection in Patients After Pediatric Liver Transplantation

**DOI:** 10.3389/fped.2022.915795

**Published:** 2022-06-03

**Authors:** Zhuyuan Si, Chong Dong, Chao Sun, Kai Wang, Wei Zhang, Weiping Zheng, Xinzhe Wei, Wei Gao, Zhongyang Shen

**Affiliations:** ^1^First Central Clinic Institute, Tianjin Medical University, Tianjin, China; ^2^Organ Transplantation Center, Tianjin First Central Hospital, Tianjin, China; ^3^Key Laboratory of Transplantation, Chinese Academy of Medical Sciences, Tianjin First Central Hospital, Tianjin, China; ^4^Tianjin Key Laboratory of Organ Transplantation, Tianjin First Central Hospital, Tianjin, China

**Keywords:** late-onset acute cellular rejection, pediatric liver transplantation, nomogram, individualized prediction, risk factors

## Abstract

**Background:**

Late-onset acute cellular rejection (LACR) is a special type of acute rejection (AR) only rarely studied after pediatric liver transplantation (pLT). Our study aimed to explore the influencing factors of LACR after pLT and establish a nomogram to provide an individualized prediction of LACR after pLT.

**Materials and Methods:**

Data from 640 children who underwent pLT at Tianjin First Central Hospital from January 2016 to December 2019 were collected as part of this retrospective study. The nomogram was then established through the results of the multivariable analysis.

**Results:**

Forty-one patients experienced LACR > 1 ≤ 2 years after pLT. Cold ischemia time, donor-specific antibodies (DSAs), and tacrolimus concentration were independent influencing factors, and a nomogram was established with an AUC value of 0.834 (95% confidence interval, 0.755–0.912). Ten-fold cross-validation showed that the accuracy of the nomogram was about 76%. Sixty-three patients experienced LACR > 2 years after pLT. Child–Pugh grade, cold ischemic time, DSAs, early acute cellular rejection, and tacrolimus concentration were independent influencing factors, and a nomogram was established with an AUC value of 0.827 (95% confidence interval, 0.774–0.881). Ten-fold cross-validation showed that the accuracy of the nomogram was about 80.9%.

**Conclusion:**

We established nomograms to predict the incidence of LACR > 1 ≤ 2 and > 2 years after pLT, respectively. The verification results showed that nomograms had good accuracy and clinical practicability.

## Introduction

At present, liver transplantation (LT) is the most effective method for the treatment of end-stage liver disease in children. In the early stage of LT, allograft rejection is one of the most common causes of graft loss. However, with the introduction of new medications and the improvement of treatment regimens, graft loss caused by acute rejection has become less common (1). With the development of transplantation technology, the survival time of patients after transplantation has gradually increased (2), but the concentration of immunosuppressants in patients gradually tends to decrease, and medication compliance is also uneven across patients. As such, late-onset acute cellular rejection (LACR) confirmed by pathology has attracted increasing attention (3).

The pathology of LACR is similar to early acute cellular rejection, in that it is characterized by mononuclear portal vein inflammation, subendothelial inflammation, and inflammatory bile duct injury (4). LACR after pediatric liver transplantation (pLT) can lead to graft loss, reduced survival rate, chronic rejection, and deterioration of prognosis (5), and it often occurs in the period of low immunosuppression after LT (6).

The incidence rate of LACR varies from 6.7–40% (6–8). LACR has been proven to be harmful to long-term prognosis; thus, the prevention and management of LACR are of great clinical importance in pLT (5). Studies have confirmed that LACR has a significant effect on graft degradation and subsequent graft failure in older children (9). One study showed that an increase in the standard deviation of tacrolimus blood concentration will increase the risk of LACR, indicating the need for more rigorous and prospective monitoring of tacrolimus blood concentrations (10). However, the tacrolimus concentrations predicting LACR at various stages after pLT have not been elucidated, yet it would be very useful to determine the occurrence, development, and risk factors of LACR after pLT. Therefore, we retrospectively evaluated the risk factors of LACR and explored the validity of an individualized evaluation prediction model to predict and monitor the occurrence of LACR.

## Materials and Methods

### Patient Selection

A retrospective analysis of consecutive pLTs (patients aged ≤ 14 years) performed between January 2016 and December 2019 at Tianjin First Central Hospital, Tianjin, China, was conducted. The inclusion criteria were as follows: (i) the pLT was the first pLT; (ii) clinical and follow-up data were available; (iii) the follow-up duration was >1 year. In addition, the endpoints of follow-up were death, graft loss, or LACR. For those patients who did not reach follow-up endpoints, the final date of data collection was October 31, 2021. This study was approved by Tianjin First Central Hospital Medical Ethics Committee (approval number: 2021N151KY).

### Definition of Late-Onset Acute Cellular Rejection

All cases of acute rejection (AR) were diagnosed by biopsy. According to the proportion of LACR in each stage, cases of AR that occurred >1 year after pLT were defined as instances of LACR, and those that occurred ≤1 year after pLT were defined as instances of early acute cellular rejection.

### Immunosuppression

Patients received an immunosuppressive regimen consisting of tacrolimus and methylprednisolone when ≤6 years old but received an immunosuppressive regimen consisting of tacrolimus, methylprednisolone, and mycophenolate mofetil (MMF) when >6 years old. The dose of MMF was 10 mg/kg body weight/day, and the medication was taken twice a day for 6 months, then discontinued. The initial dose of tacrolimus was 0.1–0.3 mg/kg body weight/day, and the target blood concentration maintenance level was 7–10 ng/mL at 1–3 months after surgery, 5–8 ng/mL at 3–6 months after surgery, 4–6 ng/mL at 6–12 months after surgery, 3–5 ng/mL at 1–2 years after surgery, and 2–4 ng/mL at >2 years after surgery, respectively.

As the target concentration of tacrolimus was different between 1–2 years and >2 years, patients were divided into two groups according to their follow-up time. The selection criteria of tacrolimus concentration were as follows: (i) the last recorded tacrolimus concentration was 1 month before the diagnosis of LACR in patients with LACR and (ii) the last recorded tacrolimus concentration was 1 month before the last follow-up visit in patients without LACR.

There was a significant statistical difference in the follow-up time between the LACR group and no LACR group among those patients with a follow-up time of >2 years. Therefore, cases with no statistical difference in follow-up time were screened by 1:4 propensity score matching so that the concentration of tacrolimus was comparable. The comparison control groups were in the same age groups as the LACR, but were not diagnosed with LACR.

### Statistical Analysis

The variables were screened by univariate and multivariate analyses, and variables with a *P*-value of <0.05 in the multivariate analysis were included in the nomogram. The area under the receiver operating characteristic (ROC) curve (AUC) calculated by bootstrapping was used to evaluate discriminative ability. The AUC varied from 0.5–1.0, and 0.5 represented a random chance, and 1.0 represented a perfect fit. Generally, an AUC of >0.7 indicates that the estimation is reasonable. The calibration curve was used to evaluate the consistency of the nomogram. Decision curve analysis (DCA) was used to evaluate the clinical benefit and practicability of the nomogram constructed using the selected variables.

Ten-fold cross-validation (CV) was used to assess the accuracy of the prediction and to address the model fit. The kappa value was used to measure the stability of the nomogram. Generally, it is believed that the nomogram has acceptable consistency if the kappa value is >0.2.

Categorical variables are expressed as frequencies and proportions. Continuous variables that conformed to the positive distribution are expressed as mean and standard deviation (SD) values, while continuous variables that did not conform to the positive distribution are expressed as median and interquartile range values. The chi-squared test or Fisher’s exact test was used to analyze the categorical variables, when appropriate. The Mann–Whitney *U*-test or independent-samples *t*-test was used to assess the continuous variables, when appropriate. Binary logistic regression was used for univariable and multivariable analyses. Statistical analyses were conducted using the R version 4.1.1 (www.r-project.org; R Foundation for Statistical Computing, Vienna, Austria). *P* < 0.05 was considered to be statistically significant.

## Results

### Study Population

Among the children who underwent pLT, 640 met the inclusion criteria. There were 288 males (45%) and 352 females (55%); and the median age at operation was 7.63 months (range, 6.10–11.6 years). Among the primary diseases, 577 cases were biliary atresia (90.2%), 16 cases were cholestasis (2.5%), three cases were liver tumors (0.5%), nine cases were Alagille syndrome (1.4%), and 35 cases were other diseases (5.5%). These were similar in patients without LACR and those with LACR. A total of 437 patients received living donor liver transplantation (LDLT) (68.3%), 203 patients received non-LDLT (31.7%). LACR occurred in 104 (16.3%) of the 640 patients.

### Number of Patients With Acute Rejection in Different Periods

**FIGURE 1:**
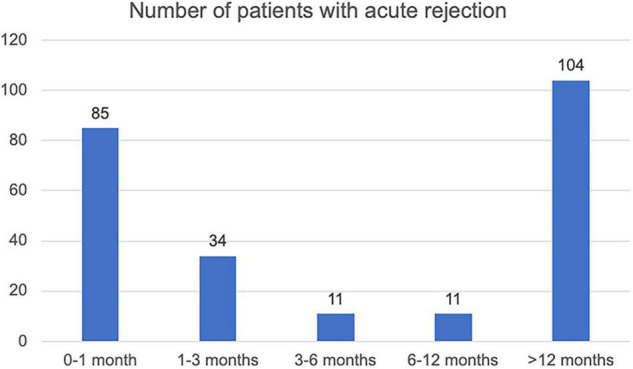
Number of patients who experienced AR in different periods.

The number of patients with AR in each period is shown in Figure 1. Thirty-five percent of patients developed AR ≤ 1 month after surgery. Fourteen percent of patients developed AR 1–3 months after surgery. AR occurred in 4.5% of patients between 3–6 months and 6–12 months after surgery, respectively. Finally, 42% of patients developed AR > 1 year after surgery.

### Characteristics and Univariate Analysis of Patients Followed for >1 ≤ 2 Years

One hundred thirty-three recipients were followed for >1 ≤ 2 years. Of these 133 patients, 41 had LACR and 92 did not have LACR. There was no significant difference in follow-up time between the two groups (*P* > 0.05). The clinical characteristics and results of the univariable analysis are summarized in Table 1. In the clinical information, there were significant differences in terms of age at surgery, donor type, cold ischemia time, DSAs, and tacrolimus concentration between the two groups (*P* < 0.05). No significant difference was observed in other variables (*P* > 0.05). The ratio of LDLT to Not LDLT for Not LACR is 0.46 and the LDLT to Not LDLT for LACR is 1.28. Thus, ∼2.78 higher likelihood of LACR with Not LDLT.

**TABLE 1:** Clinical characteristics and univariable analysis of patients followed for >1 ≤ 2 years.

Variables	Not LACR	LACR	*P*-value
Total	92	41	
Follow-up time (median [IQR])	617.5 [552, 675]	671 [491, 723]	0.314
Gender (%)			0.623
Male	44 (47.8)	17 (41.5)	
Female	48 (52.2)	24 (58.6)	
Age at surgery (median [IQR])	8.12 [6.56, 14.90]	9.37 [7.50, 27.77]	0.021
Diagnosis (%)			0.332
Biliary atresia	83 (90.2)	34 (82.9)	
Cholestasis	5 (5.4)	2 (4.9)	
Liver tumor	1 (1.1)	0	
Alagille syndrome	1 (1.1)	1 (2.4)	
Others	2 (2.2)	4 (9.8)	
BMI (median [IQR])	16.42 (1.89)	15.83 (1.56)	0.080
PELD score (median [IQR])	18 [7, 24]	14 [7, 25]	0.368
Child-pugh grade (%)			0.860
A	13 (14.1)	7 (17.1)	
B/C	79 (85.9)	34 (82.9)	
Donor type (%)			0.013
LDLT	63 (68.5)	18 (43.9)	
Not LDLT	29 (31.5)	23 (56.1)	
Graft weight (median [IQR])	241 [214.75, 271]	270 [216, 350]	0.062
GRWR [mean (SD)]	3.35 (1.01)	3.20 (1.21)	0.464
Cold ischemia time (median [IQR])	97 [75, 175.25]	278 [85, 600]	0.002
Anhepatic time (median [IQR])	43 [38, 50.25]	46 [39, 55]	0.264
Blood compatibility (%)			0.600
Identical	63 (68.5)	31 (75.6)	
Compatible	23 (25.0)	7 (17.1)	
Incompatible	6 (6.5)	3 (7.3)	
DSAs (%)			<0.001
Negative	85 (92.4)	21 (51.2)	
Positive	7 (7.6)	20 (48.8)	
Early acute cellular rejection (%)			0.186
No	76 (82.6)	29 (70.7)	
Yes	16 (17.4)	12 (29.3)	
PTLD (%)			1.000
No	86 (93.5)	38 (92.7)	
Yes	6 (6.5)	3 (7.3)	
EBV (%)			0.770
No	24 (26.1)	9 (22.0)	
Yes	68 (73.9)	32 (78.0)	
CMV (%)			0.479
No	35 (38.0)	19 (46.3)	
Yes	57 (62.0)	22 (53.7)	
Lung infection (%)			0.701
No	73 (79.3)	32 (78.0)	
Yes	19 (20.7)	9 (22.0)	
Blood infection (%)			0.161
No	75 (81.5)	38 (92.7)	
Yes	17 (18.5)	3 (7.3)	
Tacrolimus concentration (median [IQR])	3.80 [3.14, 4.90]	3.20 [2.60, 4.20]	0.013

*LACR, late-onset acute cellular rejection; OR, odd ratio; IQR, interquartile range; BMI, body mass index; PELD, pediatric end-stage liver disease; LDLT, living donor liver transplantation; GRWR, graft recipient weight ratio; SD, standard deviation; DSAs, donor-specific antibodies; PTLD, posttransplant lymphoproliferative disorders; EBV, Epstein-Barr virus; CMV, cytomegalovirus.*

### Multivariable Analysis of Patients Followed Up for 2 Years

After univariable analysis, age at surgery, donor type, cold ischemia time, DSAs, and tacrolimus concentration were included in the multivariable logistic regression analysis (Table 2). Multivariable analysis demonstrated that the occurrence of LACR was significantly correlated with cold ischemia time, DSAs, and tacrolimus concentration (*P* < 0.05).

**TABLE 2:** Results of multivariable logistic regression analyses.

Variables	Multivariable analysis	*P*-value
	OR (95% CI)	
Age at surgery	1.021 (0.997–1.045)	0.085
Donor type		0.384
LDLT	1	
Not LDLT	0.482 (0.093–2.491)	
Cold ischemia time	1.005 (1.001–1.008)	0.014
DSAs		<0.001
Negative	1	
Positive	10.641 (3.468–32.651)	
Tacrolimus concentration	0.612 (0.424–0.885)	0.009

*OR, odd ratio; CI, confidence interval; LDLT, living donor liver transplantation; DSAs, donor-specific antibodies.*

### Establishment and Verification of the Nomogram of Patients Followed for >1 ≤ 2 Years

**FIGURE 2:**
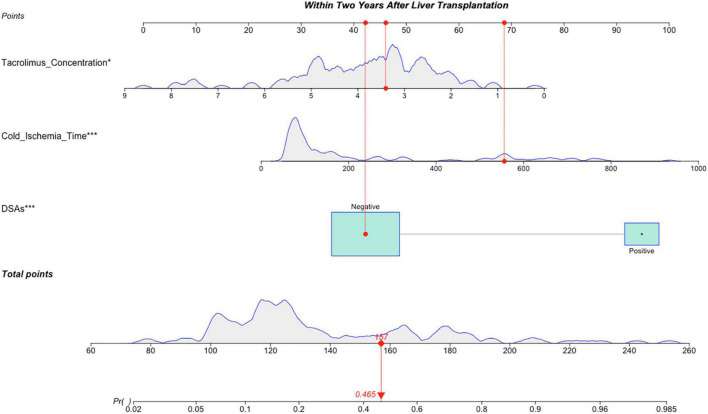
A constructed nomogram for LACR prediction >1 ≤ 2 years after pLT. The “example” patient’s cold ischemia time was 556 min, DSAs were negative and tacrolimus concentration at 1–2 years after transplantation was 3.4 ng/mL. Density plot of tacrolimus concentration, cold ischemia time and total points shows their distributions. For DSAs, the distribution is reflected by the size of the box. The degree of influence was arranged from top to bottom. In order to use the nomogram, the specific points (red dots) of individual patients are located on each variable axis according to the clinical datum. The red dots of each variable are reflected upward on the Points axis; the sum of these points (157) is located on the Total Points axis, and a line is drawn down to the Probability axis to determine the probability of LACR. In this example, 0.465. LACR, late-onset acute cellular rejection; DSAs, donor-specific antibodies. *p-value < 0.05; **p-value < 0.01; ***p-value < 0.001.

**FIGURE 3:**
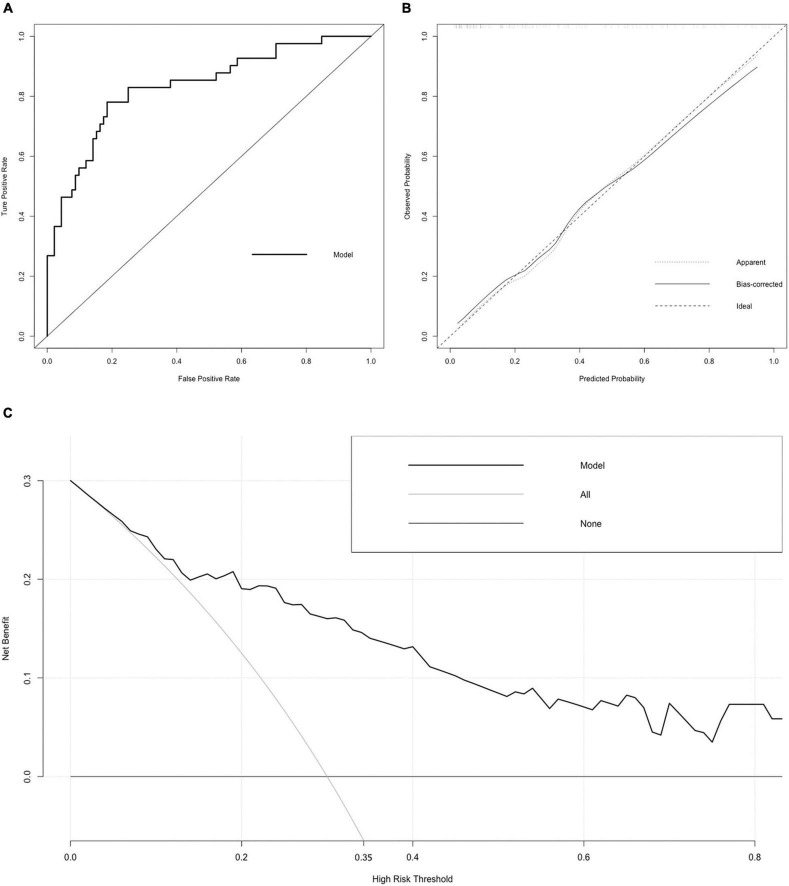
Receiver operating characteristic (ROC) curve, calibration curve, and DCA curve of the nomogram. **(A)** ROC curve. The AUC of the nomogram was 0.834 (95% CI = 0.755–0.912). **(B)** Calibration curve. The black dotted line represents the ideal reference line, where the predicted probability matches the observed probability. The solid line is calculated by bootstrapping (resampling: 1,000) and represents the performance of nomogram. The closer the solid line is to the black dotted line, the more accurate the model predicts the incidence of LACR. **(C)** DCA curve. The dark black dotted line indicates that assuming that all patients do not have LACR, the net benefit rate is 0. The light black dotted line indicates that assuming that LACR occurs in all patients, the net benefit rate is a backslash with a negative slope, and the “high risk” threshold is 0.35. The solid line is the decision curve the nomogram, reflecting the relationship between threshold probability and net benefit. The closer the curve is to the upper right, the greater the net benefit under the same threshold probability. ROC curve, receiver operating characteristic curve; AUC, area under curve; CI, confidence interval; LACR, late-onset acute cellular rejection; DCA, decision curve analysis.

Variables with significant statistical differences in the multivariate analysis were included in the construction of the nomogram (Figure 2). The evaluation performances were assessed using the ROC curve (Figure 3A). The AUC value was 0.834 (95% CI, 0.755–0.912). The AUC value was > 0.7 for the prediction of LACR, indicating favorable discrimination by the nomogram. The calibration curve was used to evaluate the difference between the predicted value and the actual value (Figure 3B), and it showed high consistency. The DCA curve was used to evaluate the clinical benefit of the nomogram (Figure 3C). CV was used to assess the accuracy of the prediction and to address the model fit. Through 10-fold CV, the nomogram possessed an accuracy of 0.760, with a kappa value of 0.406, demonstrating good accuracy and consistency. The algorithm of the nomogram based on patients followed for >1 ≤ 2 years was converted to a dynamic nomogram using the following website: https://sizhuyuan.shinyapps.io/WithinTwoYearsAfterLiverTransplantation/.

### Characteristics and Univariate Analysis of Patients Followed for >2 Years

A total of 507 recipients were followed for >2 years. Because there were significant statistical differences in the follow-up time of these patients (*P* < 0.05), a 1:4 propensity score matching was conducted according to whether LACR occurred and the follow-up time. There was no significant statistical difference in the follow-up time after propensity score matching (*P* > 0.05) among 63 patients with LACR and 252 patients without LACR. The clinical characteristics and results of the univariable analysis are summarized in Table 3. In the clinical information, there were significant differences in terms of age at surgery, Child–Pugh grade, donor type, cold ischemia time, anhepatic time, DSAs, early acute cellular rejection, and tacrolimus concentration between the two groups (*P* < 0.05). No significant difference was observed in other variables (*P* > 0.05). The ratio of LDLT to Not LDLT for Not LACR is 0.44 and the LDLT to Not LDLT for LACR is 0.91. Thus, ∼2.07 higher likelihood of LACR with Not LDLT.

**TABLE 3:** Clinical characteristics and univariable analysis of patients followed for >2 years.

Variables	Not LACR	LACR	*P*-value
Total	252	63	
Follow up time (median [IQR])	916 [817.25, 1129.25]	887 [785.6, 1114.5]	0.477
Gender (%)			0.977
Male	115 (45.6)	28 (44.4)	
Female	137 (54.4)	35 (55.6)	
Age at surgery (median [IQR])	7.30 [5.95, 10.60]	8.93 [6.72, 18.04]	0.002
Diagnosis (%)			0.795
Biliary atresia	229 (90.9)	55 (87.3)	
Cholestasis	3 (1.2)	1 (1.6)	
Liver tumor	1 (0.4)	0	
Alagille syndrome	5 (2.0)	1 (1.6)	
Others	14 (5.6)	6 (9.5)	
BMI (median [IQR])	16.30 [15.00, 17.60]	16.00 [14.95, 17.15]	0.495
PELD score (median [IQR])	17.5 [12, 22]	14 [3.5, 23.5]	0.162
Child-pugh grade (%)			0.002
A	25 (9.9)	16 (25.4)	
B/C	227 (90.1)	47 (74.6)	
Donor type (%)			0.016
LDLT	175 (69.4)	33 (52.4)	
Not LDLT	77 (30.6)	30 (47.6)	
Graft weight (median [IQR])	250 [203, 283.5]	240 [210, 300]	0.678
GRWR [mean (SD)]	3.37 (0.98)	3.34 (1.23)	0.834
Cold ischemia time (median [IQR])	104.5 [77, 186]	132 [85.5, 515.5]	0.007
Anhepatic time (median [IQR])	44 [38, 52.25]	50 [42.5, 58.5]	0.003
Blood compatibility (%)			0.299
Identical	171 (67.9)	43 (68.3)	
Compatible	41 (16.3)	14 (22.2)	
Incompatible	40 (15.9)	6 (9.5)	
DSAs (%)			<0.001
Negative	217 (86.1)	40 (63.5)	
Positive	35 (13.9)	23 (36.5)	
Early acute cellular rejection (%)			0.015
No	206 (81.7)	42 (66.7)	
Yes	46 (18.3)	21 (33.3)	
PTLD (%)			0.194
No	238 (94.4)	56 (88.9)	
Yes	14 (5.6)	7 (11.1)	
EBV (%)			0.926
No	76 (30.2)	18 (28.6)	
Yes	176 (69.8)	45 (71.4)	
CMV (%)			0.474
No	101 (40.1)	29 (46.0)	
Yes	151 (59.9)	34 (54.0)	
Lung infection (%)			0.602
No	198 (78.6)	52 (82.5)	
Yes	54 (21.4)	11 (17.5)	
Blood infection (%)			0.290
No	223 (88.5)	52 (82.5)	
Yes	29 (11.5)	11 (17.5)	
Tacrolimus concentration (median [IQR])	3.30 [2.50, 4.30]	2.30 [1.75, 3.10]	<0.001

*LACR, late-onset acute cellular rejection; OR, odd ratio; IQR, interquartile range; BMI, body mass index; PELD, pediatric end-stage liver disease; LDLT, living donor liver transplantation; GRWR, graft recipient weight ratio; SD, standard deviation; DSAs, donor-specific antibodies; PTLD, posttransplant lymphoproliferative disorders; EBV, Epstein-Barr virus; CMV: cytomegalovirus.*

### Multivariable Analysis of Patients Followed for >2 Years

After univariable analysis, age at surgery, Child–Pugh grade, donor type, cold ischemia time, anhepatic time, DSAs, early acute cellular rejection, and tacrolimus concentration were included in the multivariable logistic regression analysis (Table 4). Multivariable analysis demonstrated that the occurrence of LACR was significantly correlated with Child–Pugh grade, cold ischemia time, DSAs, early acute cellular rejection, and tacrolimus concentration (*P* < 0.05).

**TABLE 4:** Results of multivariable logistic regression analyses.

Variables	Multivariable analysis	*P*-value
	OR (95% CI)	
Age at surgery	0.998 (0.978–1.018)	0.831
Child-pugh grade		0.013
A	1	
B/C	0.300 (0.116–0.776)	
Donor type		0.598
LDLT	1	
Not LDLT	0.750 (0.258–2.184)	
Cold ischemia time	1.003 (1.000–1.005)	0.037
Anhepatic time	1.013 (0.993–1.034)	0.209
DSAs		0.007
Negative	1	
Positive	2.727 (1.308–5.682)	
Early acute cellular rejection		0.003
No	1	
Yes	3.126 (1.487–6.573)	
Tacrolimus concentration	0.354 (0.243–0.514)	<0.001

*OR, odd ratio; CI, confidence interval; LDLT, living donor liver transplantation; DSAs, donor-specific antibodies.*

### Establishment and Verification of the Nomogram of Patients Followed for >2 Years

**FIGURE 4:**
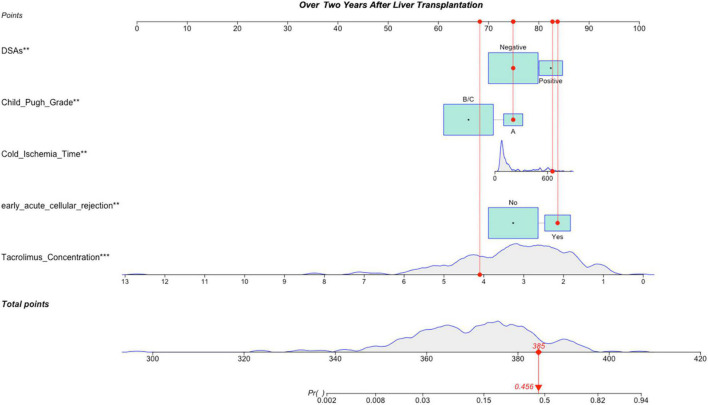
A constructed nomogram for LACR prediction >2 years after pLT. The “example” patient’s child-pugh grade was A, cold ischemia time was 657 min, DSAs were negative, early acute cellular rejection was yes and tacrolimus concentration over 2 years after transplantation was 4.1 ng/mL. Density plot of tacrolimus concentration, cold ischemia time and total points shows their distributions. For category variables, their distributions are reflected by the size of the box. The degree of influence was arranged from top to bottom. In order to use the nomogram, the specific points (red dots) of individual patients are located on each variable axis according to the clinical datum. The red dots of each variable are reflected upward on the Points axis; the sum of these points (385) is located on the Total Points axis, and a line is drawn down to the Probability axis to determine the probability of LACR. In this example, 0.456. LACR, late-onset acute cellular rejection; DSAs, donor-specific antibodies. *p-value < 0.05; **p-value < 0.01; ***p-value < 0.001.

**FIGURE 5:**
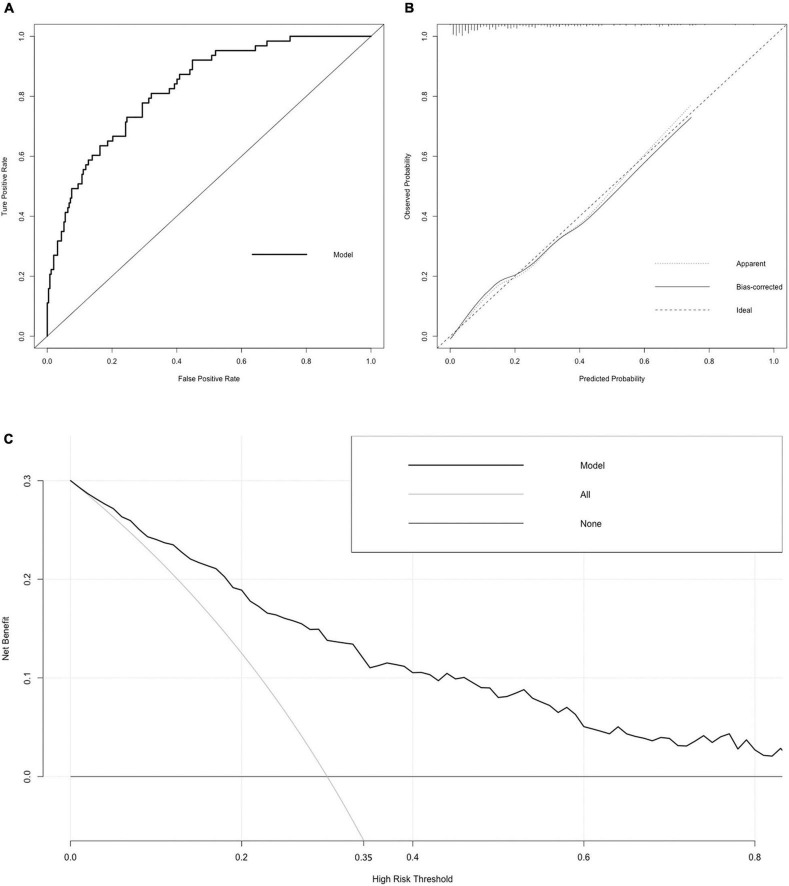
Receiver operating characteristic (ROC) curve, calibration curve, and DCA curve of the nomogram. **(A)** ROC curve. The AUC of the nomogram was 0.827 (95% CI = 0.774–0.881). **(B)** Calibration curve. The black dotted line represents the ideal reference line, where the predicted probability matches the observed probability. The solid line is calculated by bootstrapping (resampling: 1,000) and represents the performance of nomogram. The closer the solid line is to the black dotted line, the more accurate the model predicts the incidence of LACR. **(C)** DCA curve. The dark black dotted line indicates that assuming that all patients do not have LACR, the net benefit rate is 0. The light black dotted line indicates that assuming that LACR occurs in all patients, the net benefit rate is a backslash with a negative slope, and the “high risk” threshold is 0.35. The solid line is the decision curve the nomogram, reflecting the relationship between threshold probability and net benefit. The closer the curve is to the upper right, the greater the net benefit under the same threshold probability. ROC curve, receiver operating characteristic curve; AUC, area under curve; CI, confidence interval; LACR, late-onset acute cellular rejection; DCA, decision curve analysis.

Variables with significant statistical differences in multivariate analysis were included in the construction of the nomogram (Figure 4). The evaluation performances were assessed using the ROC curve (Figure 5A), and the AUC value was 0.827 (95% confidence interval, 0.774–0.881). Notably, the AUC value was >0.7 for the prediction of LACR, indicating favorable discrimination by the nomogram. The calibration curve was used to evaluate the difference between the predicted value and the actual value (Figure 5B), and it showed high consistency. The DCA curve was used to evaluate the clinical benefit of the nomogram (Figure 5C). CV was used to assess the accuracy of the prediction and to address the model fit. Through 10-fold CV, the nomogram revealed an accuracy of 0.809, with a kappa value of 0.306, demonstrating good accuracy and consistency. The algorithm of the nomogram based on patients followed for >2 years was converted to a dynamic nomogram using the following website: https://sizhuyuan.shinyapps.io/OverTwoYearsAfterLiverTransplantation/.

## Discussion

The incidence of early acute rejection has improved significantly in the past three decades due to the improvement of immunosuppressive regimens (11). However, the incidence of LACR has not improved accordingly. There are few studies on LACR after LT in the world, and there are even fewer studies on LACR after pLT. There is no evidence that the incidence rate of LACR varies after pediatric and adult LT. According to the existing findings, it is generally believed that the occurrence of LACR is related to non-adherence, weaning of immunosuppression medications, and HLA mismatches at class II, and it may also be related to the maturation of the immune system (12).

The purpose of this study was to establish an individualized predictive model of LACR after pLT. Using a dynamic nomogram, we can conveniently and quickly display the probability of LACR for different patients, based on their individual parameters, and also predict the subsequent probability of LACR in those same patients after adjusting the tacrolimus dosing, as well as, other parameters. All of this can provide for personalized patient care, as guidance to the physician care providers.

Of the 640 patients enrolled in this retrospective study, 104 developed LACR, with a 16.25% incidence rate, consistent with the range of 6.7–40% in reported incidence rates among other centers. The division time of LACR is completely subjective. Most researchers used 6 months as the cutoff value to divide LACR and early acute cellular rejection. Through statistical analysis, our center found that the number of cases of acute cellular rejection decreased gradually with the extension of time, with 35% of cases occurring ≤1 month and 14% of cases occurring between 1 and 3 months after surgery, but the proportion of acute cellular rejection accounted for about 42% of the total number of acute cellular rejections after 1 year. Therefore, we thought that it was more meaningful to divide LACR and early acute cellular rejection based on a 1-year cutoff.

In our study, there was a significant statistical difference between LACR and early acute cellular rejection >2 years after pLT. The risk of LACR was significantly increased in patients with early acute cellular rejection (odds ratio, 3.126; *P* = 0.003). El Ters et al. (13) showed that early acute cellular rejection was not a single acute event but instead triggered a persistent alloimmune response. Similar events may occur again several years after the acute event and eventually lead to long-term graft injury and graft loss. These findings are consistent with our observations.

It is generally believed that the risks associated with LACR include transplantation for autoimmune disease, poor adherence, and immunosuppressive changes (7). Meanwhile, the potential consequences of poor medication adherence include increased medical costs, hospitalization, allograft loss, and death, and they pose a major dilemma for medical service providers responsible for allocating scarce organs. However, there is no universally accepted tool to monitor medication compliance. When LACR occurs, it can usually be treated by increasing the tacrolimus concentration. However, the extent to which tacrolimus can effectively reduce the occurrence of LACR, especially for different patients at different times, and how to control the individualized tacrolimus concentration level have not been studied. Our study not only proved that the tacrolimus concentration was an independent factor affecting the occurrence of LACR at different times after pLT but also established an individualized prediction model including the tacrolimus concentration that can effectively predict the occurrence probability of LACR after transplantation and after adjusting the tacrolimus concentration.

Ischemia–reperfusion injury during transplantation is characterized by delayed graft function, which can upregulate a large number of cytokines and adhesion molecules and increase the expression of HLA class II molecules on the graft endothelium to increase the patient’s susceptibility to AR after transplantation (12). This is consistent with our study finding that cold ischemia time is an independent risk factor for LACR. The incidence rate of AR increased significantly in children who had DSAs after 1 year. This is consistent with our results. Wozniak et al. (14) showed that DQ DSAs are strongly associated with LACR.

This study also has some limitations. First, although the prediction model established in this study showed good accuracy and consistency through internal verification, its effect has not been verified by external data. Second, the prediction model established in this study is only limited to rejection. When altering the tacrolimus concentration, it only observed the change in the incidence of LACR, without considering the change in the incidence of complications, such as infection, posttransplant lymphoproliferative disorders, metabolic syndrome, renal function injury, and so on.

## Conclusion

We established nomograms to predict the incidence rate of LACR > 1 ≤ 2 and > 2 years after pLT. The independent factors of the two groups were cold ischemia time, DSAs, and tacrolimus concentration and Child–Pugh grade, cold ischemia time, DSAs, early acute cellular rejection, and tacrolimus concentration, respectively. The verification results showed that the nomograms had good accuracy and clinical practicability.

## Data Availability Statement

The original contributions presented in the study are included in the article/Supplementary Material, further inquiries can be directed to the corresponding authors.

## Ethics Statement

Written informed consent was obtained from the minor(s)’ legal guardian/next of kin for the publication of any potentially identifiable images or data included in this article.

## Author Contributions

WG and ZSh participated in research design. ZSi and WG participated in the writing of the manuscript. ZSi, CD, CS, KW, WZ, and WPZ participated in the performance of the research. ZSi and XW participated in data analysis. All authors contributed to the article and approved the submitted version.

## Conflict of Interest

The authors declare that the research was conducted in the absence of any commercial or financial relationships that could be construed as a potential conflict of interest.

## Publisher’s Note

All claims expressed in this article are solely those of the authors and do not necessarily represent those of their affiliated organizations, or those of the publisher, the editors and the reviewers. Any product that may be evaluated in this article, or claim that may be made by its manufacturer, is not guaranteed or endorsed by the publisher.
